# Protein tyrosine phosphatase PTPRO represses lung adenocarcinoma progression by inducing mitochondria-dependent apoptosis and restraining tumor metastasis

**DOI:** 10.1038/s41419-023-06375-x

**Published:** 2024-01-05

**Authors:** Yuan Dai, Shuangshuang Shi, Hongda Liu, Hong Zhou, Wenqiu Ding, Chenyang Liu, Linling Jin, Weiping Xie, Hui Kong, Qun Zhang

**Affiliations:** 1 Department of Respiratory and Critical Care Medicine, The First Affiliated Hospital of Nanjing Medical University, Nanjing, 210029 Jiangsu China; 2 Department of Respiratory Medicine, Jiangsu Province Official Hospital, Nanjing, China; 3 Department of General Surgery, The First Affiliated Hospital of Nanjing Medical University, Nanjing, 210029 China

**Keywords:** Non-small-cell lung cancer, Apoptosis

## Abstract

Emerging evidence indicates that protein activities regulated by receptor protein tyrosine phosphatases (RPTPs) are crucial for a variety of cellular processes, such as proliferation, apoptosis, and immunological response. Protein tyrosine phosphatase receptor type O (PTPRO), an RPTP, has been revealed as a putative suppressor in the development of particular tumors. However, the function and the underlying mechanisms of PTPRO in regulating of lung adenocarcinoma (LUAD) are not well understood. In this view, the present work investigated the role of PTPRO in LUAD. Analysis of 90 pairs of clinical LUAD specimens revealed significantly lower PTPRO levels in LUAD compared with adjacent non-tumor tissue, as well as a negative correlation of PTPRO expression with tumor size and TNM stage. Survival analyses demonstrated that PTPRO level can help stratify the prognosis of LUAD patients. Furthermore, PTPRO overexpression was found to suppress the progression of LUAD both in vitro and in vivo by inducing cell death via mitochondria-dependent apoptosis, downregulating protein expression of molecules (Bcl-2, Bax, caspase 3, cleaved-caspase 3/9, cleaved-PARP and Bid) essential in cell survival. Additionally, PTPRO decreased LUAD migration and invasion by regulating proteins involved in the epithelial-to-mesenchymal transition (E-cadherin, N-cadherin, and Snail). Moreover, PTPRO was shown to restrain JAK2/STAT3 signaling pathways. Expression of PTPRO was negatively correlated with p-JAK2, p-STAT3, Bcl-2, and Snail levels in LUAD tumor samples. Furthermore, the anti-tumor effect of PTPRO in LUAD was significant but compromised in STAT3-deficient cells. These data support the remarkable suppressive role of PTPRO in LUAD, which may represent a viable therapeutic target for LUAD patients.

## Introduction

Tyrosine phosphorylation is a crucial regulator of various human physiological phenomena, including cellular activation, gene transcription, and environmental homeostasis. This process is tightly regulated by the coordinated action of protein tyrosine kinases (PTKs) and protein tyrosine phosphatases (PTPs) [1, 2]. While numerous researchers focus on PTKs, which have dominated the cancer target therapeutics sphere. Increasing articles have reported that PTPs play a role in certain physiological and pathological activities by regulating tyrosine dephosphorylation, which is an opposing but equally significant mechanism as PTKs [3, 4]. Protein tyrosine phosphatase receptor type O (PTPRO), one of the receptor-type PTPs (RPTPs), was first found in renal glomerular epithelial cells [5]. As a transmembrane protein and expressed in several parenchymal and inflammatory cells (including lung, liver, breast, macrophages, and lymphocytes) [6], PTPRO has been linked to both elemental biological processes and development of inflammatory diseases, such as embryogenesis [7], osteoclast function [8], immune response [9], neuron differentiation [10, 11], atherosclerosis [6], lung injury [12, 13] and hepatitis [14]. Increasing studies demonstrated the critical functions of PTPRO in tumor suppression of certain cancer types. For example, PTPRO exhibits tumor-suppressive properties in chronic lymphocytic leukemia through negative regulating of B-cell receptor (BCR) signaling [15]. In the case of breast cancer, PTPRO promoter methylation is a prognostic factor for HER2-positive patients and suppresses ERBB2-driven breast cancer growth by promoting ERBB2 dephosphorylation and endocytotic degradation [9, 16]. Moreover, PTPRO-mediated autophagy inhibits tumorigenesis in hepatocellular carcinoma (HCC) [17], and PTPRO downregulation is associated with an IL-6-driven increase in PD-L1 expression in monocytes and macrophages [18]. In addition, by increasing immunological infiltrates, PTPRO may serve as a therapeutic target in pancreatic cancer [19]. However, its role and mechanisms in lung cancer remain unknown.

Lung cancer is the most prevalent cause of cancer-related death, contributing to the highest morbidity and mortality rates worldwide [20–22]. Lung cancer is expected to account for 12% of all new cancer diagnoses in 2023 [22]. The two primary histological subtypes of lung cancer are non–small-cell lung cancer (NSCLC) and small-cell lung cancer (SCLC). NSCLC, which accounts for about 80% of all lung cancer cases, is classified into two main subtypes: lung adenocarcinoma (LUAD, ~48% of NSCLC) and lung squamous cell carcinoma (LSCC, ~28%) [21]. With significant advances in LUAD treatment such as targeted therapies and immunotherapies, LUAD survival has increased remarkably in the last decade [23]. The development of targeted therapeutics for lung cancer greatly benefits from abnormal tyrosine phosphorylation catalyzed by RPTKs, such as the Epidermal Growth Factor Receptor (EGFR). On the other hand, RPTPs-mediated tyrosine dephosphorylation counterbalance oncogenic tyrosine kinase signaling [24, 25]. Nevertheless, limited research focuses on the roles of RPTPs, including PTPRO, in LUAD. In the present investigation, we examine the patterns of PTPRO expression in LUAD and its prognostic significance. Moreover, we performed systematic tests via in vitro and in vivo strategies to explore the inhibitory effect and the underlying molecular mechanism of PTPRO in LUAD, which could serve as a novel anticancer target.

## Methods

### In silico test

PTPRO mRNA levels in lung cancer and normal tissues were retrieved from the TCGA and GEO datasets (GEO19188, GEO19804). GSEA was performed on TCGA cohorts using software from Broad Institute (http://software.broadinstitute.org/gsea/index.jsp). Low- or high-PTPRO expression was distinguished using the mean cutoff based on their risk score expression. The differentially expressed genes (DEGs) between these two groups were subjected to gene set enrichment analysis (GSEA) and KEGG pathway analysis to investigate possible differences in biological processes and signaling pathways.

### Immunohistochemical (IHC) staining

The protein expression profile of PTPRO in lung adenocarcinomas and adjacent nontumorous lung tissues was assessed using a commercially available tissue microarray (Shanghai Outdo Biotech, Shanghai, China) with the IHC method. LUAD biopsy were acquired from ten patients in Nanjing Medical University First Affiliated Hospital. Briefly, the tissue microarray and LUAD tissues were subjected to the following procedures: deparaffinization, rehydration, antigen retrieval by microwave, inactivation of endogenous peroxidase, primary antibody incubation, secondary antibody incubation, stain development, and counterstaining [26]. Two independent pathologists blinded to patients’ information analyzed IHC results based on staining intensity and proportion of positively stained cells.

### Cells and transient transfection

The human lung adenocarcinoma cell lines HCC827, PC9, and H1975 were employed in this investigation and cultured as previously described [21]. GeneChem (Shanghai, China) helped insert the PTPRO coding region into the GV230 vector. Transient transfection was performed using Lipofectamine3000 following the manufacturer’s protocol. Blank vector GV230 served as a negative control. Transfection efficiency was validated by Western blotting. Each experiment was repeated at least three times independently.

### Lentivirus and CRISPR plasmids

The lentivirus vector GV492 (Ubi-MCS-3FLAG-CBh-gcGFP-IRES-puromycin) containing PTPRO coding region and empty vector were purchased from Genechem (Shanghai, China). For lentivirus infection, cells were initially cultured in 6-well plates at a concentration of 3 × 10^4^ cells per well for a duration of 24 h. Subsequently, they were subjected to transduction with an optimal quantity of prepared lentiviral vectors as previously detailed [21]. Following a post-transfection incubation period of 3–4 days, cells were subjected to selective pressure using a puromycin-containing growth medium, rendering them suitable for subsequent experimentation.

The preparation of CRISPR-Cas9/gRNA plasmid DNA followed established procedures, as detailed in previous studies [27]. In brief, gRNAs against STAT3 were designed and annealed into LentiCRISPRv2 plasmids (Supplementary Table 1). Positive clones harboring the gRNA-encoding DNA sequences were rigorously validated through DNA sequencing. Lipofectamine 3000 was employed for plasmid transfection, and the assessment of transfection efficacy was subsequently conducted through Western blot.

### Western blotting (WB)

Protein levels in cells and tissues were measured using WB. Cells or isolated tissues were lysed with RIPA buffer (Beyotime, Nantong, China) supplemented with protease inhibitor cocktail (Roche, Basel, Switzerland) and PMSF (Roche), and total protein concentrations were determined using the Bradford strategy. Subsequently, 20–30 µg protein was separated by electrophoresis, followed by membrane transfer, blocking, primary antibody incubation, secondary antibody incubation, and immunodetection [28]. Detailed antibody information was listed in Supplementary Table 2. Immunoblotting results from three independent repeats were semi-quantified using ImageJ Plus Software.

### 2,5-diphenyl-2H-tetrazolium bromide (MTT) assay

The MTT assay was used to evaluate cell proliferation. Briefly, transfected cells were seeded into 96-well plates at a density of 3000 cells/well. The cells were then cultured for 1, 2, 3, and 4 days, respectively. At each time point, 10 µl MTT solution was added to each well to achieve a working concentration of 0.45 mg/ml. Following another 3-h incubation, the medium was removed and 100 µl solubilization solution was added to each well to dissolve MTT crystals. Absorbance was recorded at 570 nm using a microplate reader (SpectraMax iD5). Each experiment was repeated three times.

### Colony formation

Colony formation was performed to assess the survival ability of single cells. Transfected cells were seeded in 6-well plates at a density of 500 cells/well. Cells were cultured for 2 weeks in an 5% CO_2_ incubator at 37 °C, and subsequently fixed, and stained with crystal violet. Colony numbers were photographed and counted. Each experiment was repeated three times.

### Flow cytometry

Cells were trypsinized 48 h after transfection and subjected to an Annexin V/PI assay using a flow cytometric method to evaluate apoptosis, as previously described [21]. Experiments on apoptosis were conducted in triplicate.

### JC-1 staining

The JC-1 assay kit (Beyotime Biotech, Nantong, China) was used to assess mitochondrial membrane potential of LUAD cells, following the manufacturer’s protocol [29]. Briefly, the JC-1 (1×) working solution was added in the prepared cells and incubated at 37 degrees for 20 min and then photographed using a fluorescence microscope. The shift in fluorescence emission from red to green indicated the decline in mitochondrial membrane potential, a characteristic feature in the early stages of apoptosis.

### Mitochondrial permeability transition pore (MPTP) assay

The opening of MPTP is a critical event leading to cell death, which was assessed using an MPTP kit (Beyotime Biotech, Nantong, China) following the manufacturer’s protocol [29]. Briefly, cells on the coverslip were washed and labeled for 30 min in a dark room at 37 degrees and examined under a Leica Thunder DMi8 microscope. Diminished green fluorescence indicates increased MPTP opening.

### Transwell assay

Cell migration and invasion potentials were evaluated using the Transwell assays [30]. Briefly, transfected cells were seeded into the transwell insert (8 μm pore size) at a density of 30,000 cells/well for migration test. The upper chamber was filled with 2% FBS medium, while the lower chamber was filled with 15% FBS medium. Cells that migrated to the bottom side of the insert were fixed and stained for counting after 24 h. The invasion assay was performed similarly to the migration assay, except that the seeding density was 80,000 cells/well and the transwell insert was pre-coated with Matrigel (Cat. 356234, Corning, NY, USA).

### Subcutaneous xenograft and tail vein injection mice models

The subcutaneous xenograft model and tail vein injection model were developed using BALB/c nude male mice (4–5 weeks old), respectively. Lentivirus-mediated stable transfected cells (~1 × 10^7^) were subcutaneously injected into nude mice. Tumor length and width were recorded every 5 days using a vernier caliper. The tumor volume was calculated using the formula: length × width × width × 0.5. Mice were sacrificed and xenografts were excised 20 days later.

The same amount of transfected cells was injected into the tail vein to establish the tail vein injection model, and mice were cultured for 4 weeks before being sacrificed. The lungs of mice were then resected to assess metastatic tumor.

### Statistics

Data were processed using SPSS 22.0, Graphpad Prism 7.0, and R 4.0 Software. Overall survival time was defined as the period between disease diagnosis and death or the last follow-up date. A Chi-square test was performed for the difference test. Kaplan–Meier and log-rank tests were used for survival analysis. The Cox hazard regression model was utilized for multivariate analysis to identify independent risk factors. Student’s *t* test and one-way ANOVA test were used to distinguish differences between groups. Two-tail *p* < 0.05 denoted statistical difference.

## Results

### Low PTPRO expression is associated with advanced TNM stage and poor prognosis of LUAD patients

**Fig. 1:**
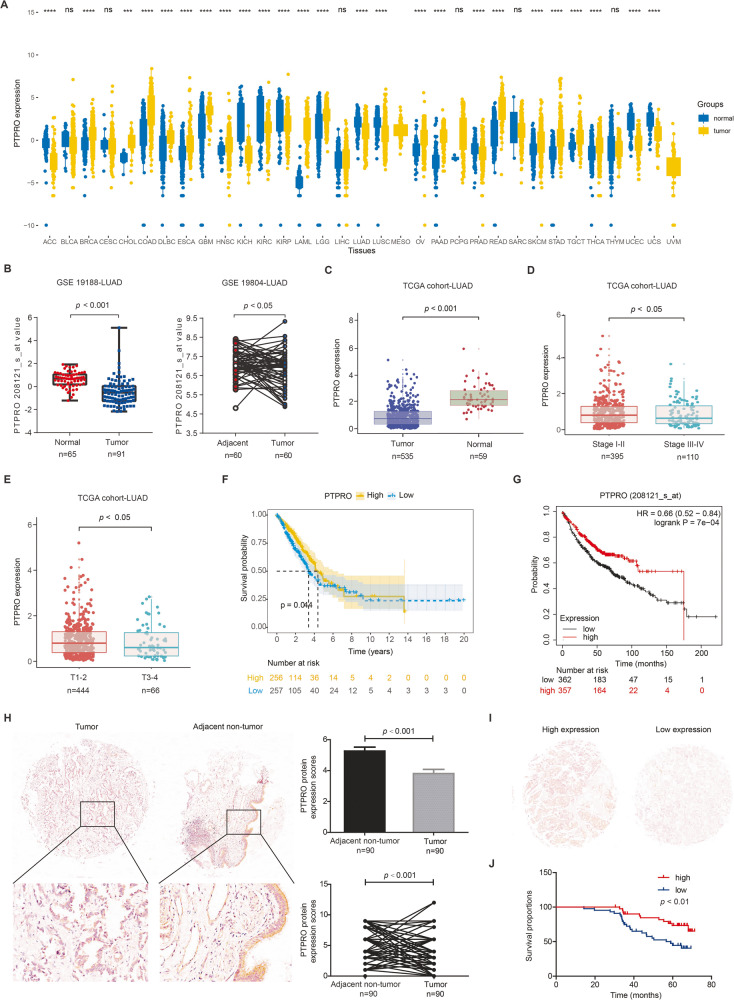
Low PTPRO expression is associated with advanced TNM stage and poor prognosis of LUAD patients. **A** Pan-cancer analysis of PTPRO gene expression in different tumor types from TCGA datasets. **B** Different PTPRO gene expression in LUAD and adjacent normal lung specimens in GEO datasets (GSE19188 and GSE 19804). **C** PTPRO gene expression difference between LUAD and normal lung tissues in TCGA cohort. **D** PTPRO gene expression in LUAD with TNM stage I–II vs. those with TNM stage III–IV in TCGA cohort. **E** PTPRO gene expression in LUAD with T stage T1–2 vs. T stage T3–4 in TCGA cohort. **F**, **G** Kaplan–Meier overall survival analyses of LUAD patients according to the gene level of PTPRO in TCGA cohort and a combined cohort (TCGA, EGA, GEO), respectively. **H** Representative IHC staining of PTPRO protein expression in paired LUAD tissues and adjacent normal specimens in microarray. Statistical comparison of PTPRO immunoreactivity scores were exhibited in the right panels. **I** Representative IHC staining of high expression and low expression of PTPRO in LUAD specimens. **J** Kaplan–Meier overall survival analysis of LUAD patients according to PTPRO protein expression level. ACC adrenocortical carcinoma, BLCA adrenocortical carcinoma, BRCA breast invasive carcinoma, CESC cervical squamous cell carcinoma and endocervical adenocarcinoma, CHOL cholangiocarcinoma, COAD colon adenocarcinoma, DLBC lymphoid neoplasm diffuse large B-cell lymphoma, ESCA esophageal carcinoma, GBM glioblastoma multiforme, HNSC head and neck squamous cell carcinoma, KICH kidney chromophobe, KIRC kidney renal clear cell carcinoma, KIRP kidney renal papillary cell carcinoma, LAML acute myeloid leukemia, LGG brain lower grade glioma, LIHC liver hepatocellular carcinoma, LUAD lung adenocarcinoma, LUSC lung squamous cell carcinoma, MESO mesothelioma, OV ovarian serous cystadenocarcinoma, PAAD pancreatic adenocarcinoma, PCPG pheochromocytoma and paraganglioma, PRAD prostate adenocarcinoma, READ rectum adenocarcinoma, SARC sarcoma, SKCM skin cutaneous melanoma, STAD stomach adenocarcinoma, TGCT testicular germ cell tumors, THCA thyroid carcinoma, THYM thymoma, UCEC uterine corpus endometrial carcinoma, UCS uterine carcinosarcoma, UVM uveal melanoma.

Pan-cancer analysis of The Cancer Genome Atlas (TCGA) cohort revealed distinguished expression of PTPRO in different tumor types (Fig. 1A). For example, PTPRO was upregulated in cholangiocarcinoma (CHOL) and colon adenocarcinoma (COAD), while downregulated in bladder cancer (BLCA) and kidney chromophobe (KICH). This may be attributed to several factors, including the complexity of cancer biology and the unique characteristics of each tumor type. The tumor microenvironment, including interactions with immune cells, stromal cells, and the extracellular matrix, can influence gene expression. The differential regulation of one gene in different tumor types is a reflection of the intricate and multifaceted nature of cancer. This once again underscores the importance of considering the specific context of each tumor type when studying gene expression patterns and their functional significance in the context of tumorigenesis.

Specifically, downregulation of PTPRO was observed in lung cancer, including adenocarcinoma and squamous cell carcinoma. TCGA, GSE19188, and GSE19804 were then used to further assess the expression of PTPRO mRNA in LUAD specimens. In all three datasets, the mRNA level of PTPRO was significantly lower in LUAD tissues than in normal or adjacent tissues (Fig. 1B, C). Moreover, the PTPRO transcript was found to be significantly lower in LUAD patients with advanced TNM stage or positive lymph node metastases (Fig. 1D, E). Subsequently, Kaplan–Meier analysis revealed that low levels of PTPRO mRNA were linked to poor overall survival in LUAD patients in the TCGA cohort alone (Fig. 1F) or a mixed cohort including GEO, EGA and TCGA datasets (Fig. 1G).

Considering that protein level may be different with mRNA level, we further evaluated PTPRO protein expression using a tissue microarray containing 90 pairs of primary LUAD and matched normal tissues. IHC experiments revealed a significant proportion of patients with lower PTPRO protein levels in tumor specimens compared with the adjacent non-tumor tissues, and the overall PTPRO staining scores were remarkably lower in the tumor than in the non-tumorous lung (Fig. 1H). To investigate the correlation between PTPRO expression, LUAD prognosis, and clinicopathological features, LUAD cases were separated into the high expression (staining score ≥4) and low expression (staining score <4) groups based on mean PTPRO IHC score (representative IHC staining was shown in Fig. 1I). Chi-square analyses revealed positive associations of PTPRO protein accumulation with small tumor size (*p* < 0.05) and early T stage (*p* < 0.05), indicating that advanced LUAD patients had relatively low protein expression levels of PTPRO in cancer tissues (Table 1), which was consistent with its mRNA levels in Fig. 1D.

**Table 1:** Characteristics of LUAD patients and their associations with PTPRO protein level.

Variable	Cases	PTPRO protein expression		*p* value
	(*n* = 86)	Low (*n* = 44)	High (*n* = 42)	
Age (years)				
<60 years	37	19	18	0.976
≥60 years	49	25	24	
Sex				
Female	42	23	19	0.514
Male	44	21	23	
Tumor diameter				
<3.0 cm	48	20	28	0.048*
≥3.0 cm	38	24	14	
Differentiation				
Well	7	4	3	0.799
Moderate	59	31	28	
Poor	20	9	11	
T stage				
T1a–T1b	29	10	19	0.035*
T1c	36	19	17	
T2	21	15	6	
N stage				
N0	54	27	27	0.664
N1	21	10	11	
N2	11	7	4	
M stage				
M0	64	31	33	0.388
M1	22	13	9	

*p < 0.05.

Bold values indicates statistically significant *p* values less than 0.05.

Similarly, according to Kaplan–Meier analysis of the microarray cohort, high PTPRO protein expression predicted favorable outcomes in LUAD patients (Fig. 1J), which was also consistent with the predictive role of its mRNA level in Fig. 1F, G. Furthermore, multivariate analyses demonstrated that low PTPRO protein level and distant metastasis can both independently predict a poor overall survival of LUAD (Table 2). These findings strongly suggested PTPRO as an independent prognostic factor for LUAD.

**Table 2:** Univariate and multivariate Cox regression analyses for the overall survival of LUAD patients.

Variable	Univariate			Multivariate		
	HR	95% CI	*p* value^a^	HR	95% CI	*p* value^b^
Age (years)			0.907			
<60 years	Reference					
≥60 years	0.961	0.491–1.879				
Sex			0.079			
Female	Reference					
Male	1.849	0.930–3.675				
Tumor diameter			0.452			
<3.0 cm	Reference					
≥3.0 cm	0.773	0.395–1.512				
Differentiation			0.799			
Well	Reference					
Moderate	1.089	0.256–4.638				
Poor	1.507	0.333–6.826				
T stage			0.693			
T1a–T1b	Reference					
T1c	1.235	0.573–2.662				
T2	0.886	0.348–2.156				
N stage			0.477			
N0	Reference					
N1	1.029	0.453–2.338				
N2	1.689	0.711–4.015				
M stage			<0.001***			<0.001***
M0	Reference		Reference			
M1	7.804	3.81–15.998		6.879	3.348–14.137	
PTPRO protein expression			0.004**			0.040*
Low	Reference		Reference			
High	0.360	0.174–0.744		0.462	0.222–0.965	

*p < 0.05; **p < 0.01; ***p < 0.001.

^a^*p* value was calculated by log-rank test.

^b^*p* value was calculated by Cox-regression test.

Bold values indicates statistically significant *p* values less than 0.05.

### PTPRO overexpression attenuates LUAD growth both in vitro and in vivo

**Fig. 2:**
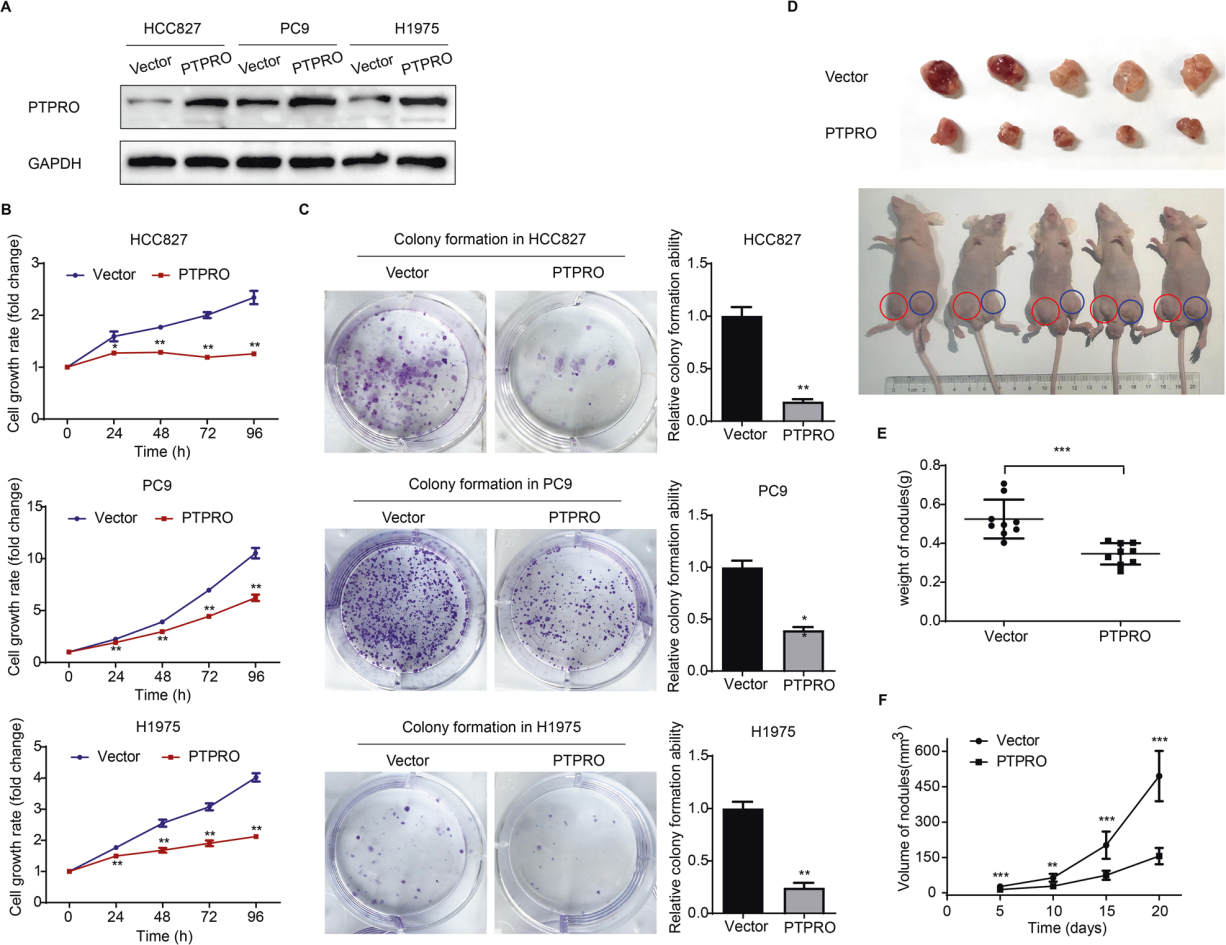
PTPRO overexpression attenuates LUAD growth both in vitro and in vivo. **A** The transfection efficiency was validated by western blotting in HCC827, PC9 and H1975 cells, after transfected with vector and PTPRO plasmids. **B** MTT assay was performed to examine the cell proliferation abilities of transfected LUAD cell lines. **C** Colony-formation assays of LUAD cells transfected with vector or PTPRO plasmids. The colonies were numbered and statistically compared in the right panel. **D** Stable transfected PC9 cells were subcutaneously injected into nude mice. After 3 weeks, mice were sacrificed, and xenografts were excised. **E** Excised tumor weight was measured as mean ± SD for all xenografts in two independently repeated experiments (*n* = 9, the other four paired xenografts are presented in Supplementary Fig. 1). **F** Tumor volume was calculated every 5 days to monitor tumor growth (*n* = 9). Data are represented as mean ± SD. *p < 0.05, **p < 0.01, ***p < 0.001.

Clinical and dataset analyses revealed that PTPRO was downregulated in LUAD; in this view, we employed GV230-PTPRO plasmid-mediated overexpression to investigate its functional role in LUAD cells. Western blot analysis revealed a significant increase following PTPRO plasmid transfection in HCC827, PC9, and H1975 cells (Fig. 2A). In a 4-day MTT assay, PTPRO-overexpression significantly decreased cell growth rate in the three LUAD cell lines (*p* < 0.01, Fig. 2B). The cell growth-inhibitory effect of PTPRO was further validated by monolayer colony formation in all the three cell lines with a substantially reduced number and size of colonies following PTPRO overexpression (Fig. 2C). These results demonstrated that PTPRO overexpression decreased LUAD cell proliferation in vitro.

The effect of PTPRO on LUAD growth was then explored in vivo. PC9 cells stably transfected with GV492-vector and GV492-PTPRO plasmids were subcutaneously injected into nude mice. Detectable tumor volumes were monitored and recorded every 5 days. The mice were sacrificed and tumor xenografts were isolated 20 days post-inoculation (Fig. 2D and Supplementary Fig. 1, two independently repeated experiments). As a result, PTPRO overexpression drastically suppressed tumor growth in nude mice, as evidenced by significantly lighter tumor weight and smaller tumor size in the PTPRO group compared with the vector group (Fig. 2E, F). These findings suggested the anti-oncogenic potential of PTPRO both in vitro and in vivo.

### PTPRO induces LUAD cell death via mitochondria-dependent apoptosis both in vitro and in vivo

**Fig. 3:**
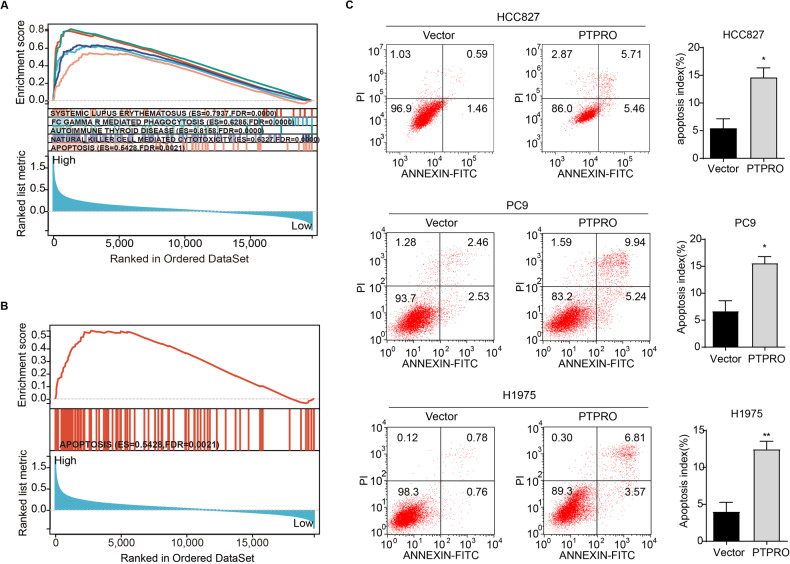
PTPRO induces cells apoptosis in LUAD. **A** The GSEA enrichment analysis of PTPRO in TCGA-LUAD cohort. **B** GSEA enrichment analysis of PTPRO regarding apoptosis pathway in LUAD. **C** Cell apoptosis was evaluated by flow cytometry strategy in HCC827, PC9 and H1975 cells between PTPRO-overexpression and vector groups. Statistical analyses were presented in the right panel. *p < 0.05, **p < 0.01.

We investigated the putative biological function pathways of PTPRO in the TCGA cohort using GSEA analysis since the previous data showed a proliferation-suppression function of PTPRO. The results indicated that PTPRO expression was linked to systemic lupus erythematosus, phagocytosis, autoimmune thyroid disease, NK cell-mediated cytotoxicity, and apoptosis (Fig. 3A, B). Therefore, the PTPRO-transfected LUAD cells were tested for apoptosis using an Annexin V/PI assay. The results showed that HCC827, PC9, and H1975 cells transfected with PTPRO had a much greater proportion of apoptosis as compared with vector-transfected cells (Fig. 3C). These findings indicated that elevated PTPRO expression could induce apoptosis of LUAD cells.

**Fig. 4:**
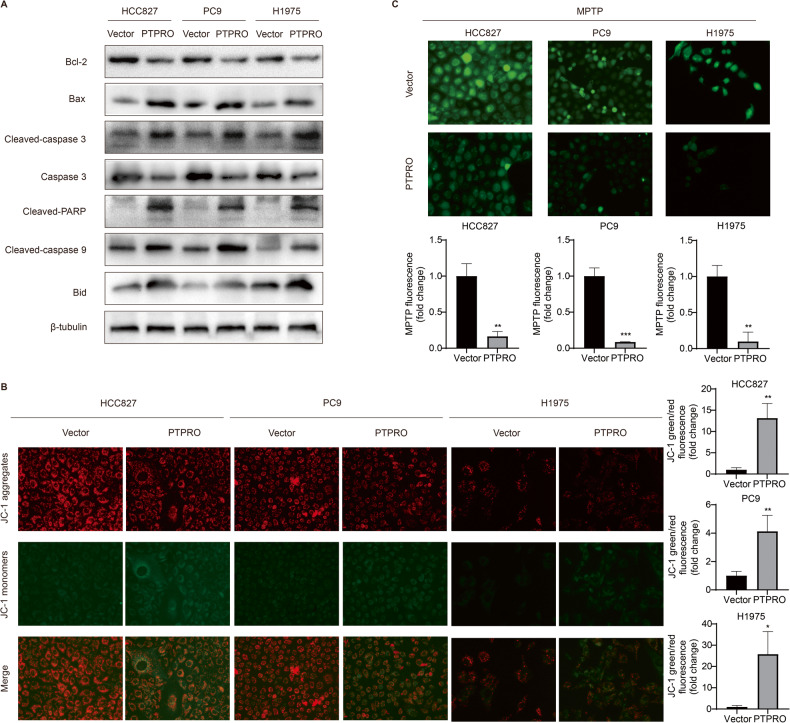
PTPRO induces mitochondria-dependent apoptosis of LUAD cells in vitro. **A** Western blotting analysis of mitochondria-dependent apoptosis pathway-related proteins in HCC827, PC9 and H1975 cells, including Bcl-2, Bax, cleaved-caspase 3, caspase 3, cleaved PARP, cleaved-caspase 9 and Bid. **B** Representative images of JC-1 staining for mitochondrial membrane potential (Δψm). Quantitative analysis of Red/Green fluorescence ratio were presented as mean ± SD (*n* = 3). **C** Representative images of MPTP opening staining (weaker green fluorescence indicated more MPTP opening), and statistically quantitative analysis of green fluorescence was demonstrated as mean ± SD (*n* = 3). *p < 0.05, **p < 0.01, ***p < 0.001.

A network of interrelated signals may induce apoptosis via two primary pathways: the death receptor pathway (extrinsic pathway) and the mitochondrial pathway (intrinsic pathway) [31]. To further illustrate the PTPRO-mediated apoptosis mechanisms, expression of apoptotic proteins was evaluated by Western blot. As shown in Fig. 4A, the protein levels of Bcl-2 and caspase 3 significantly decreased following PTPRO transfection, while the expressions of proapoptotic proteins Bax, cleaved-caspase 9, cleaved-caspase 3, cleaved-PARP and Bid markedly increased. Consistently, the gene expression of *Bid* substantially increased in PTPRO overexpression cells (Supplementary Fig. 2). Bcl-2 is an anti-apoptotic protein which inhibits all mitochondrial apoptogenic alterations, including mitochondrial membrane potential (MMP, Δψm) loss and mitochondrial permeability transition pore (MPTP) opening [32]. Consistently, our data showed that overexpression of PTPRO decreased red JC-1 fluorescence while increasing green JC-1 fluorescence, as well as significantly lowering the red/green ratio, suggesting Δψm loss in LUAD cells (Fig. 4B). The MPTP assay yielded similar results on that weaker green MPTP fluorescence was observed in PTPRO-overexpression cells, indicating a higher proportion of “active” mitochondria with MPTP opening (Fig. 4C). These findings revealed that mitochondrial dysfunction played a pivotal role in PTPRO-mediated apoptosis, which were accompanied by changes in the expression of Bcl-2 family members.

**Fig. 5:**
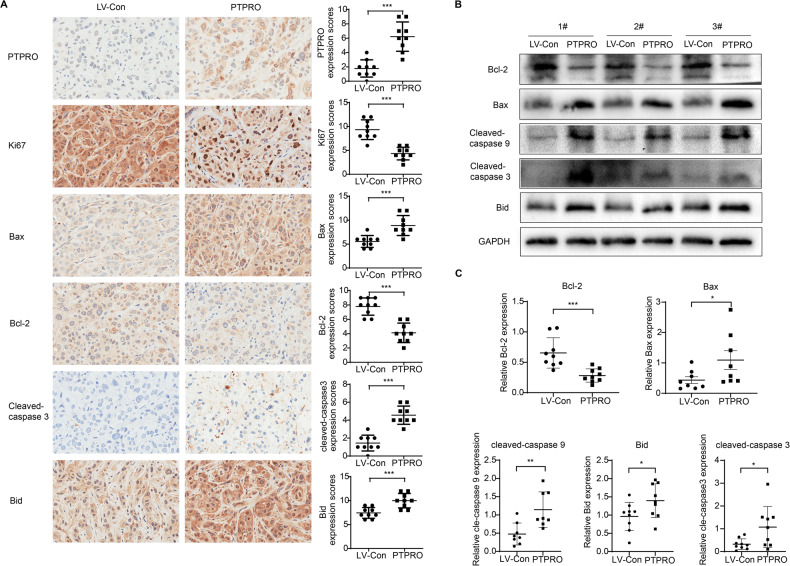
PTPRO overexpression induces mitochondria-dependent apoptosis of LUAD in vivo. **A** Representative IHC staining targeting PTPRO, Ki67, Bax, Bcl-2, cleaved-caspase 3 and Bid in the xenografts formed by subcutaneously injection of stably-transfected LUAD cells, with statistical analyses in the right panels. **B** Western blotting showed the expression of mitochondria apoptosis related proteins in resected xenografts. **C** Statistically analyses of western blotting results were indicated as mean ± SD (*n* = 9). *p < 0.05, **p < 0.01, ***p < 0.001.

IHC and Western blot were used to further examine the expression of crucial proteins linked to the mitochondrial apoptosis pathway in abovementioned isolated xenografts to validate the proapoptotic effect of PTPRO in LUAD in vivo. Ki-67 immunoreactivity was decreased in the PTPRO group compared with the vector group (Fig. 5A), indicating that PTPRO suppressed LUAD proliferation in xenografts. Bcl-2 expression was also significantly decreased following PTPRO transfection. In contrast, IHC assay revealed remarkably increments of Bax, cleaved-caspase 3 and Bid protein levels in tumors with PTPRO overexpression (Fig. 5A). Consistently, Western blot analysis revealed that PTPRO overexpression down-regulated anti-apoptotic Bcl-2 and up-regulated proapoptotic Bax, cleaved-caspase 9, cleaved-caspase 3 and Bid (Fig. 5B). Statistically analyses of western blot results were shown in Fig. 5C as the mean ± SEM (*n* = 9). These data suggested the involvement of mitochondria-dependent apoptosis pathway in PTPRO-induced apoptosis.

### PTPRO overexpression suppresses LUAD metastasis both in vitro and in vivo

**Fig. 6:**
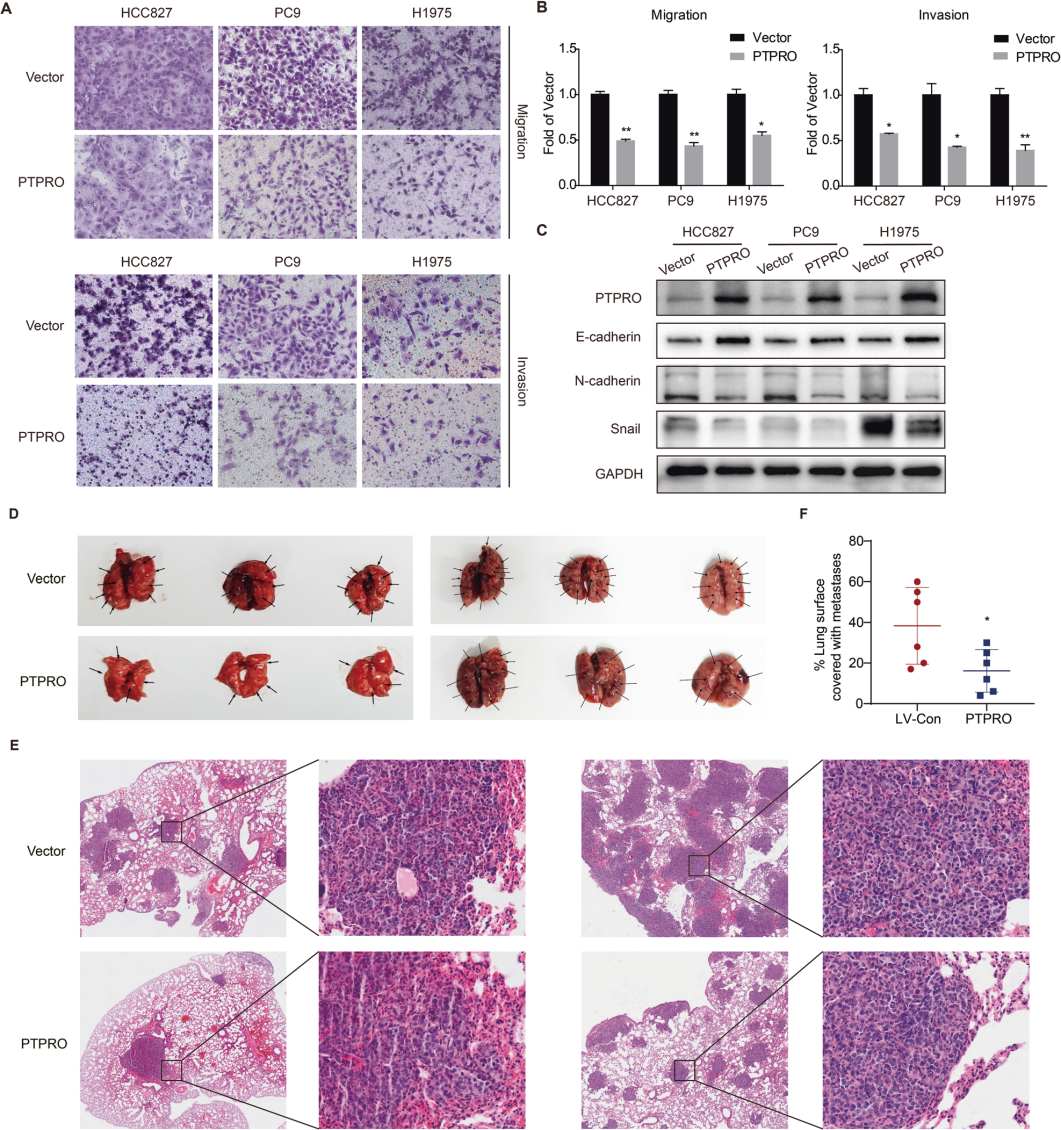
PTPRO suppresses LUAD metastasis both in vitro and in vivo. **A** Transwell assays were used to evaluate the migration and invasion abilities of HCC827, PC9 and H1975 cells transfected with vector or PTPRO plasmids. **B** Statistically analyses of transwell assays were indicated as mean ± SD (*n* = 3). **C** Western blotting demonstrated the expression of metastasis related proteins including E-cadherin, N-cadherin and Snail in LUAD cells with transfection of vector or PTPRO plasmids. **D** Stably-transfected PC9 cells were injected into tail vein, and mice were cultured for 4 weeks before sacrificed. Then mice lungs were resected to observe tumors on the lung surfaces. Two independent replicate experiments were conducted, each involving three pairs of mice. **E** Representative H&E staining of the abovementioned lung tissue slices. **F** Morphometry of lung sections (*n* = 6). *p < 0.05, **p < 0.01.

The capacity of cell migration and invasion was evaluated to further explore the inhibitory role of PTPRO in LUAD progression. Transwell assays showed overexpression of PTPRO significantly decreased the migration and invasion capacities of HCC827, PC9, and H1975 cells (Fig. 6A, B). Meanwhile, PTPRO significantly decreased the protein expression of the mesenchymal marker N-cadherin and the EMT-inducing transcription factor Snail while increasing epithelial marker E-cadherin’s level (Fig. 6C), which concur with the transwell assay results.

PC9 cells stably transfected with PTPRO or vector were injected into the tail vein of immunodeficient mice to determine whether PTPRO inhibited tumor metastasis in vivo. As a result, PTPRO group had a substantially lower number of metastatic nodules than the vector group (Fig. 6D, two independent replicated experiments). Moreover, H&E staining of mice lung slices revealed that PTPRO overexpression decreased the size and number of the metastatic nodules (Fig. 6E), as illustrated by quantifying metastatic lesions on lung sections (Fig. 6F). These results indicated that PTPRO not only reduced cancer growth viability but also suppressed the metastatic potential, highlighting its potential as an effective LUAD treatment target.

### JAK2/STAT3 signaling pathway is potentially involved in PTPRO anti-tumor activity in LUAD

**Fig. 7:**
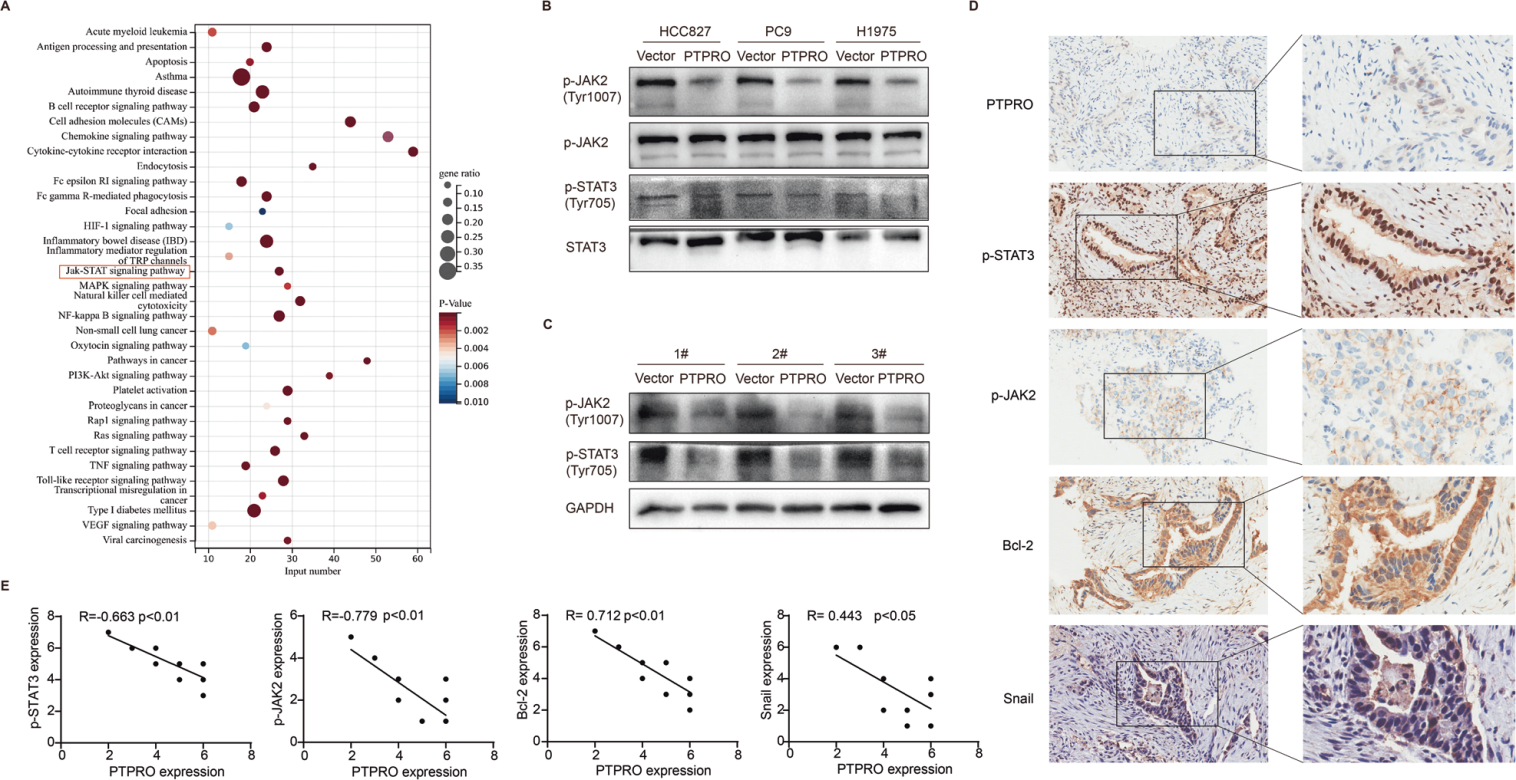
JAK2/STAT3 signaling pathway is involved in PTPRO anti-tumor activity in LUAD. **A** KEGG pathway analysis for significantly correlated genes of PTPRO in TCGA-LUAD dataset. **B** Phosphorylation levels of JAK2 and STAT3 were tested by western blotting in HCC827, PC9 and H1975 cells transfected with vector or PTPRO plasmids. **C** Western blotting analyses of p-JAK2 and p-STAT3 in mice LUAD xenografts. **D** Representative IHC staining of PTPRO, p-JAK2, p-STAT3, Bcl-2 and Snail in LUAD tissues from ten patients. **E** Spearman’s rank correlation analysis was performed to unveil the relationships between PTPRO and p-JAK2, p-STAT3, Bcl-2, Snail, respectively.

To explore the molecular mechanism of the tumor-suppression role of PTPRO in LUAD, we retrieved the PTPRO correlation genes from TCGA database for pathway enrichment analysis. KEGG pathway analysis implied the involvement of JAK/STAT signaling pathway (Fig. 7A). JAK2/STAT3 signaling has been widely implicated in mitochondria-dependent apoptosis and cancer metastasis [33, 34], thus attracted our attentions and western blotting was applied to compare JAK2/STAT3 pathway activity in PTPRO-overexpressing and control groups. According to our data, PTPRO significantly decreased phosphorylation-mediated activation of JAK2/STAT3 signaling by dephosphorylating JAK2-pY1007 and subsequent STAT3-pY705 residues in LUAD cell lines (Fig. 7B). Furthermore, decreased JAK2-pY1007 and STAT3-pY705 levels were also identified in LUAD xenografts by western blotting in the PTPRO overexpression group in vivo (Fig. 7C). Meanwhile, IHC analysis was conducted to assess the protein expressions of p-JAK2, p-STAT3, Bcl-2, Snail, and PTPRO in tissue sections obtained from ten cases of clinical lung adenocarcinoma specimens (Fig. 7D). Subsequently, Spearman’s rank correlation analysis was employed to determine the associations between these proteins. The analysis revealed significant negative correlations between PTPRO and p-JAK2, p-STAT3, Bcl-2, as well as Snail (Fig. 7E). These findings suggested a potential association of PTPRO with JAK2/STAT3 signaling pathway and its downstream molecules.

### PTPRO exerts slight anti-tumor effects in STAT3-deficient LUAD cells

**Fig. 8:**
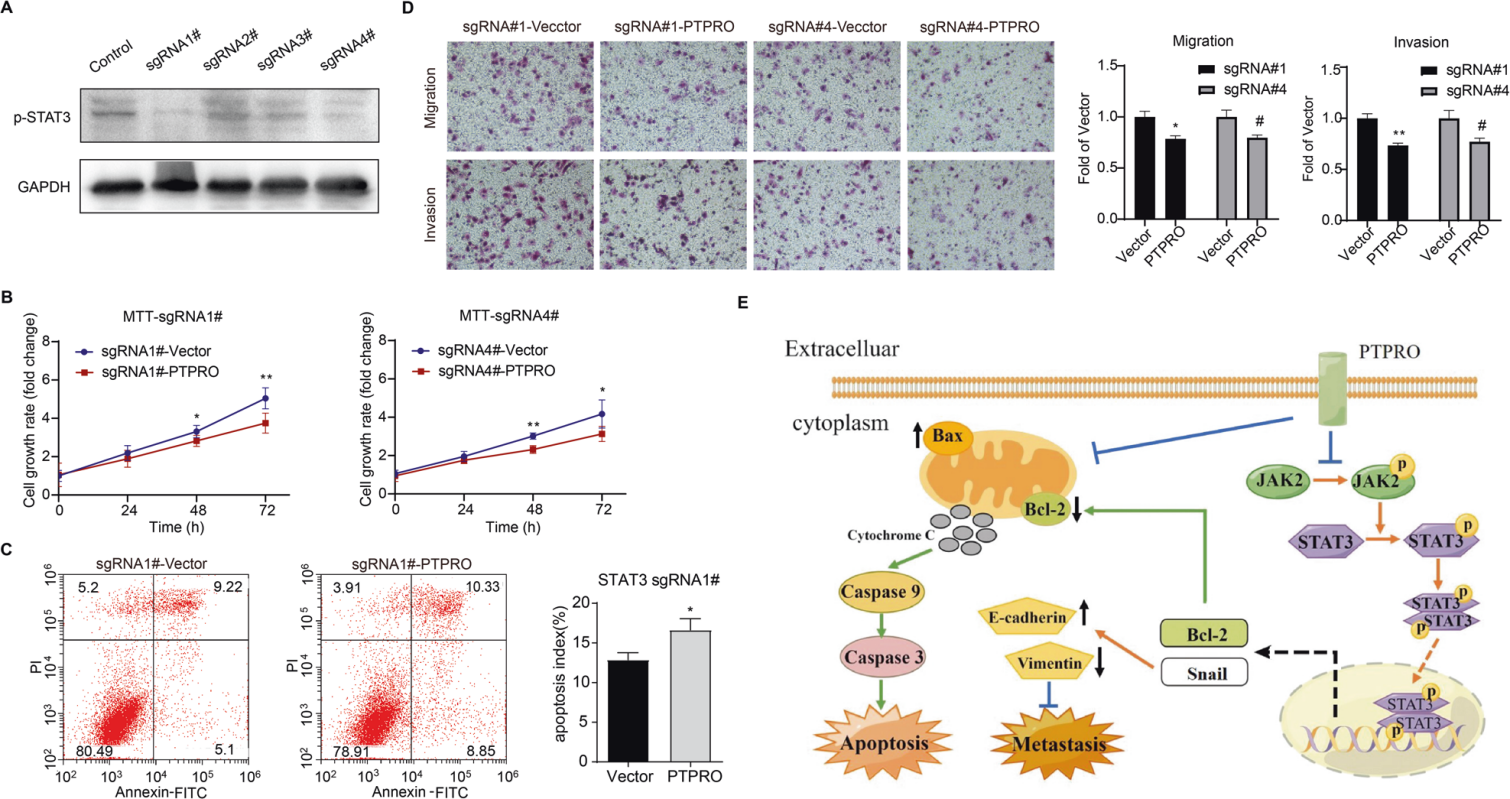
PTPRO exerts slight anti-tumor effects in STAT3-deficient LUAD cells. **A** The knockout efficiency was verified through Western blotting in PC9 cells after transfection with LentiCRISPRv2 targeting STAT3. **B** MTT assay was conducted to assess the effect of PTPRO on cell proliferation in STAT3-deficient PC9 cells. The data are represented as mean ± SD. **C** Flow cytometry strategy was used to assess cell apoptosis in STAT3-deficient cells transfected with vector or PTPRO plasmids. **D** Transwell assays were employed to evaluate the migration and invasion abilities of STAT3-deficient cells following vector or PTPRO plasmids transfection. Statistical analyses are depicted in the right panels. **E** The proposed functional mechanisms of PTPRO’s anti-tumor effects in LUAD (dotted line indicates the possible signaling pathway whereas need further validation). *p < 0.05 and **p < 0.01 indicate statistically significant by unpaired Student’s *t* test, respectively.

CRISPR/Cas9-mediated STAT3 knockout was employed to evaluate the anti-tumor effects of PTPRO in STAT3-deficient cells. Western blot analysis confirmed the successful depletion of STAT3 using CRISPR/Cas9 in PC9 cells (Fig. 8A). Subsequently, MTT assay was conducted, revealing that PTPRO overexpression can still lead to a reduction in cell viability at 48 and 72 h in STAT3-deficient cells (Fig. 8B). Nevertheless, the inhibitory effect of PTPRO on cell proliferation was compromised following STAT3 knockout, in contrast to the results observed in MTT assay presented in Fig. 2B. The Annexin V/PI assay revealed that STAT3-deficient PC9 cells transfected with PTPRO exhibited a marginally higher proportion of apoptotic cells when compared to vector-transfected cells (16.6 ± 1.99 vs. 12.9 ± 1.28, Fig. 8C). While PTPRO induced a greater degree of apoptosis in original PC9 cells (15.59 ± 1.70 vs. 6.7 ± 2.71, Fig. 3C). Consistently, although PTPRO was demonstrated to exert inhibitory effects on invasion and metastasis in STAT3-deficient PC9 cells, its inhibitory efficacy was compromised comparing to the non-STAT3-deficient PC9 cells (Figs. 6A, B and 8D).

STAT3 is a signal transducer and transcription activator that dimerized and translocated to the nucleus upon phosphorylation, regulates tumor-related genes (such as Bcl-2 and Snail) involved in cell growth, apoptosis, and migration [35]. According to published studies [34, 36], JAK2 is a well-known upstream regulator of STAT3. Taken together, PTPRO can downregulate JAK2/STAT3 phosphorylation, and inhibit oncogenic signals by suppressing expressions of Bcl-2 and Snail; these events may subsequently induce mitochondria-dependent apoptosis and suppress tumor metastasis (Fig. 8E).

## Discussion

PTPs are critical in counteracting the activity of tyrosine kinases, which are speculated to block oncogenic transformation and function as tumor suppressors. Recent evidence shows that PTPs are potential targets for cancer therapy; they play a role in cancer development and progression by dephosphorylation of tumor-related proteins and regulating signaling pathways [37]. PTPRO decrease has been linked to promoter hypermethylation in several cancer types [38–40]. Several pieces of research have suggested the potential growth-suppressor and anti-tumorigenesis properties of PTPRO, notably in hepatocellular carcinoma [18, 41], breast cancer [9, 42, 43] and colorectal tumor [44]. However, the comprehensive functions and mechanisms of PTPRO in lung adenocarcinoma (LUAD) progression are poorly understood. According to public datasets and LUAD specimens, we found that PTPRO is downregulated in LUAD, and its expression is negatively correlated with tumor size and TNM stage. Further clinical survival and multivariate analyses demonstrated that low PTPRO expression predicted a poor prognosis in LUAD patients and that it can serve as an independent risk factor in LUAD progression. Moreover, both in vitro and in vivo tests proved that PTPRO overexpression slowed the progression of LUAD and triggered cell death by the mitochondria-dependent apoptosis pathway.

Apoptosis is a well-known type of programmed cell death that plays a crucial role in the development and treatment of malignant tumors. It is activated by different extrinsic and intrinsic stimuli via two major mechanisms: the extrinsic pathway via death receptors and the intrinsic pathway via mitochondrial pathways [45]. The mitochondria-dependent apoptosis pathway is critical for tissue homeostasis and a variety of human pathologies, including tumorigenesis and cancer progression [31]. The mitochondria, as a typical executing organelle of programmed cell death, contain a variety of cell death-promoting and inhibiting factors. The mitochondria apoptosis pathway is primarily mediated by two factors following activation by various apoptotic stimuli. On the one hand, decreased Bcl-2 and increased Bax proteins integrate within the outer mitochondrial membrane (OMM), allowing for mitochondrial outer membrane permeabilization (MOMP) and the release of cytochrome c from the mitochondrial matrix into the cytoplasm. In addition, Bid accelerates mitochondrial permeabilization through the activation of Bax and inhibition of anti‐apoptotic BCL‐2 members [46]. Following that, the initiator caspase, procaspase-9, is activated into cleaved-caspase 9, culminating in the development of an apoptosome. The apoptosome stimulates the cleavage and activation of executioner caspases, such as caspase-3 and -7, which then react on various downstream effectors, eventually causing cell apoptosis [47, 48]. On the other hand, the opening of the mitochondrial permeability transition pore (MPTP) results in the shuttling of metabolic products such as ATP, ROS, and Ca^2+^, as well as matrix enlargement and rupture of the outer membrane [49, 50]. In the present investigation, protein analysis demonstrated that PTPRO downregulated anti-apoptotic Bcl-2 and upregulated pro-apoptotic Bax, resulting in mitochondria-mediated apoptosis and LUAD cell death.

STAT3 (Signal transducer and activator of transcription 3), a DNA-binding transcription factor and a site of convergence for the majority of initiated oncogenic pathways, plays a role in the pathogenesis of cancers, including apoptosis and metastasis [34]. STAT3 is activated transiently and tightly regulated in normal cells. Nonetheless, abnormal JAK2-mediated STAT3 activation has been identified in the onset and progression of various tumors [51]. B6, for example, induced apoptosis in breast cancer cells by constitutively inhibiting STAT3 phosphorylation through allosteric interaction with JAK2, implying that JAK2 is essential for STAT3 activation [52]. Inhibiting JAK2/STAT3 signaling pathway activation was found to decrease pancreatic cancer growth and induce apoptosis both in vivo and in vitro [53]. A previous study found that acylglycerol kinase overexpression augmented JAK2/STAT3 sustained activation, promoting tumorigenicity of esophageal squamous cell carcinoma (ESCC) [54]. Moreover, PTPRO dephosphorylated JAK2 and downregulated JAK2/STAT3 signaling in hepatocellular carcinoma [41]. Our study confirmed that the inhibitory efficacy of PTPRO on LUAD growth and metastasis were compromised in STAT3-deficient LUAD cells, unlike the strong inhibitory effects observed in non-STAT3-deficient cells. These results suggested that the depletion of STAT3 might partially counteract the anti-tumor effect of PTPRO, providing valuable insights into the interplay between PTPRO and JAK2/STAT3 pathway in cancer progression. Besides, these results indicate the existence of other possible mechanisms contributing to PTPRO’s anti-tumor role. Based on our results and prior research, we concluded the mechanism as followings: PTPRO suppresses the phosphorylation and activation of JAK2/STAT3 signaling pathway, inducing LUAD apoptosis by modulating the Bcl-2 family and boosting mitochondrial membrane potential (Δψm) loss as well as MPTP opening.

Aside from rapid proliferation and decreased apoptosis, metastasis is also a vital life-threatening component in the progression of LUAD. Recent findings have emphasized the potent oncogenic function of JAK2/STAT3 signaling in tumor invasion and metastasis, emphasizing the inhibition of JAK2/STAT3 pathway as a potential therapy in malignancies [55]. For example, colorectal cancer-derived mesenchymal stem cells promoted EMT and increased the migration and invasion of colorectal cancer by activating IL-6/JAK2/STAT3 signaling [56]. Brusatol inhibited human laryngeal squamous carcinoma migration and invasion presumably by blocking JAK2/STAT3 signaling [57]. In gastric cancer cells, single stranded interacting protein 1 (RBMS1) was discovered to activate the JAK2/STAT3 downstream signaling pathway after binding to the transcription factor MYC, subsequently increasing cancer migration and invasion [58]. Moreover, Snail was confirmed to be one of the downstream molecules of JAK2/STAT3 in promoting tumor metastasis [59, 60]. In this study, protein tyrosine phosphatase PTPRO consistently decreased JAK2/STAT3 tyrosine dephosphorylation and blocked downstream signaling, reducing LUAD migration and invasion. Although the above events may be independent instead of subsequent, our data suggest that PTPRO may be a viable therapeutic target for LUAD.

### Supplementary information

Reproducibility checklist

Supplementary materials

Original Data File

## Data Availability

The publicly data are provided in the article or Supplementary Materials. The datasets generated during the current study are available from the corresponding author upon reasonable request.
