# Supplementary material for: Protein tyrosine phosphatase PTPRO represses lung adenocarcinoma progression by inducing mitochondria-dependent apoptosis and restraining tumor metastasis
Source: Cell Death Dis. 2024 Jan 5;15(1):11. doi: 10.1038/s41419-023-06375-x (PMC10770368; doi:10.1038/s41419-023-06375-x)
Supplement: Supplementary file 2 — Supplementary materials [file 41419_2023_6375_MOESM2_ESM.docx]

**Supplemental Tables**

**Table S1.** sgRNA sequences against human STAT3

| Name | Sequence (5’→ 3’) |
| --- | --- |
| STAT3-sgRNA 1# | CACCGGCAGGAAGCGGCTATACTGC |
| STAT3-sgRNA 2# | CACCGGGAACAGATGCTCACTGCGC |
| STAT3-sgRNA 3# | CACCGCTACAGTGACAGCTTCCCAA |
| STAT3-sgRNA 4# | CACCGGAAGGCGTGATTCTTCCCAC |

**Table S2.** Antibodies and chemicals in this study.

| **Antibodies** | **Cat.** | **Supplier** |
| --- | --- | --- |
| PTPRO | 67000-1-Ig | Proteintech (for Western blot) |
| PTPRO | HPA034525 | Sigma (for IHC) |
| Bcl-2 | 15071S | Cell Signaling Technology |
| Bax | sc-20067 | Santa Cruz |
| Cleaved-caspase 3 | 9664S | Cell Signaling Technology |
| Caspase 3 | 9662S | Cell Signaling Technology |
| Cleaved-caspase 9 | 7237S | Cell Signaling Technology |
| Cleaved PARP | 2581S | Cell Signaling Technology |
| E-cadherin | 14472S | Cell Signaling Technology |
| N-cadherin | 13116S | Cell Signaling Technology |
| Snail | 3879S | Cell Signaling Technology |
| p-JAK2 | 4406S | Cell Signaling Technology |
| JAK2 | sc-390539 | Santa Cruz |
| p-STAT3 | 9145S | Cell Signaling Technology |
| STAT3 | sc-8019 | Santa Cruz |
| β-Tubulin | 2128S | Cell Signaling Technology |
| Bid | 2002 | Cell Signaling Technology |
| GAPDH | HRP-60004 | Proteintech |
| **Chemicals** | **Cat.** | **Supplier** |
| Lipo3000 | L3000015 | Invitrogen |

**Supplemental Figures**

**Figure S1**


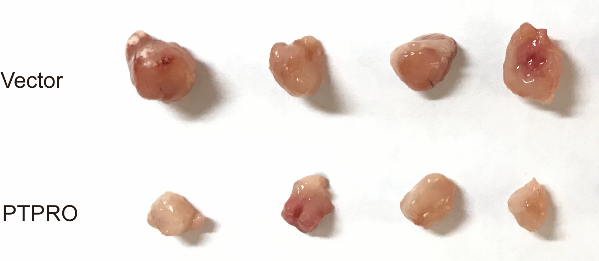


**Figure S1. Replicated xenografts in vivo.** Stable transfected PC9 cells were subcutaneously injected into nude mice. After three weeks, mice were sacrificed, and xenografts were excised. Tumor weight and tumor volume were was measured as mean±SD for all animals in figure 2.

Figure S2


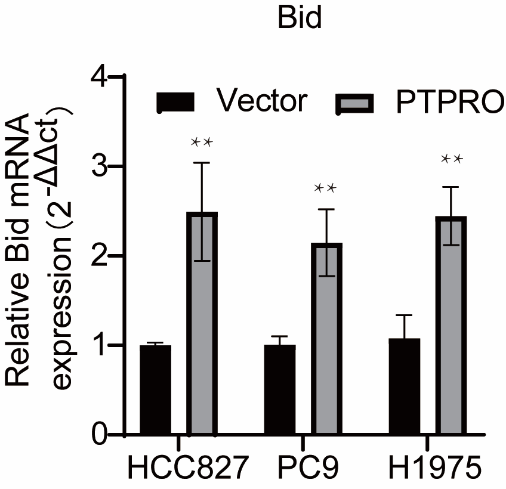


**Figure S2. mRNA expression of Bid.** qRT-PCR analysis of Bid expression in HCC827, PC9 and H1975 cells after transfection of Vector or PTPRO plasmids. ***p*＜0.01
