# Supplementary material for: Protein tyrosine phosphatase PTPRO represses lung adenocarcinoma progression by inducing mitochondria-dependent apoptosis and restraining tumor metastasis
Source: Cell Death Dis. 2024 Jan 5;15(1):11. doi: 10.1038/s41419-023-06375-x (PMC10770368; doi:10.1038/s41419-023-06375-x)

**Fig.2** PTPRO


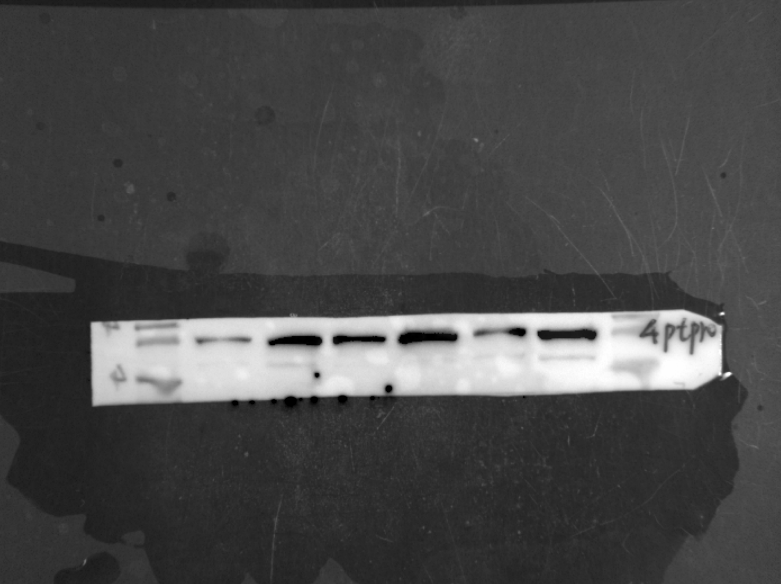


**GAPDH**


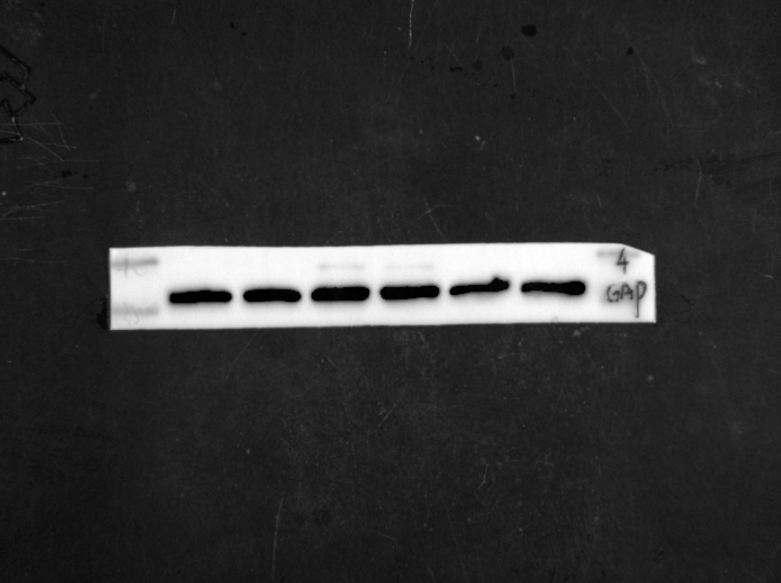


#

#

**Fig. 4** Bcl-2


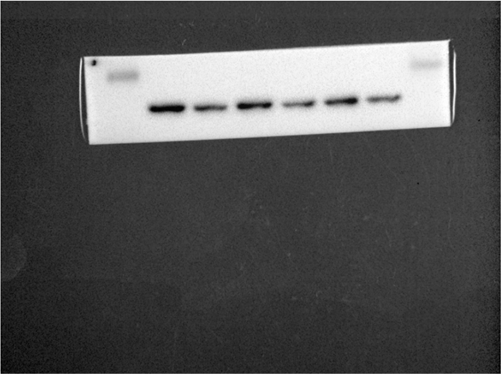


HCC827 PC9

C P C P C P

H1975

Bax


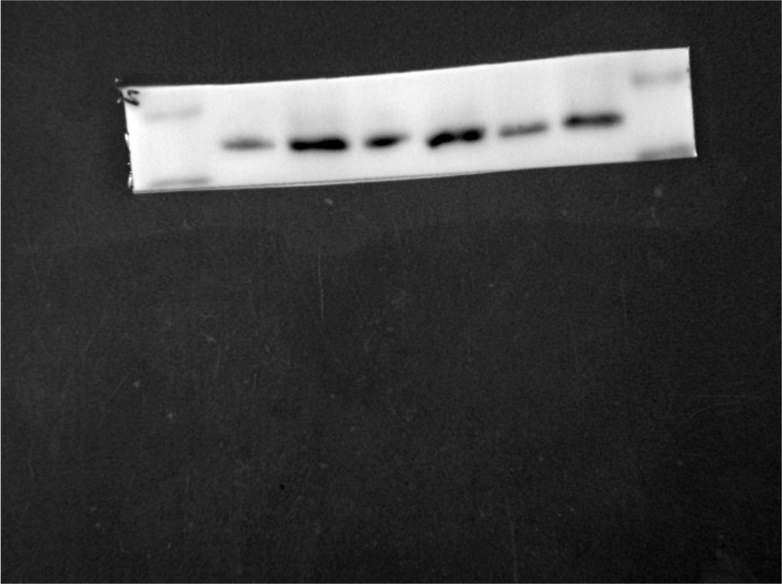


#


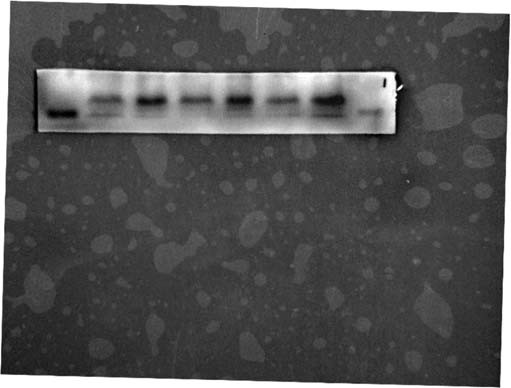
Cleaved-caspase3

Caspase 3


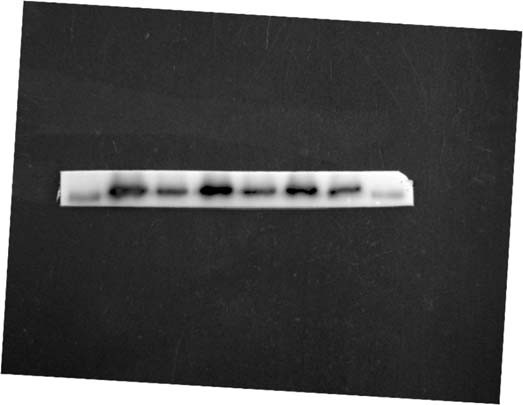


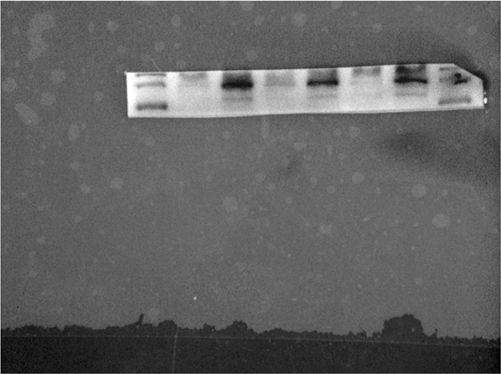
Cleaved-PARP

Cleaved-caspase9


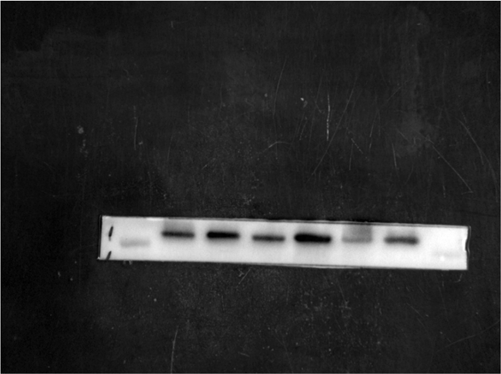


Bid


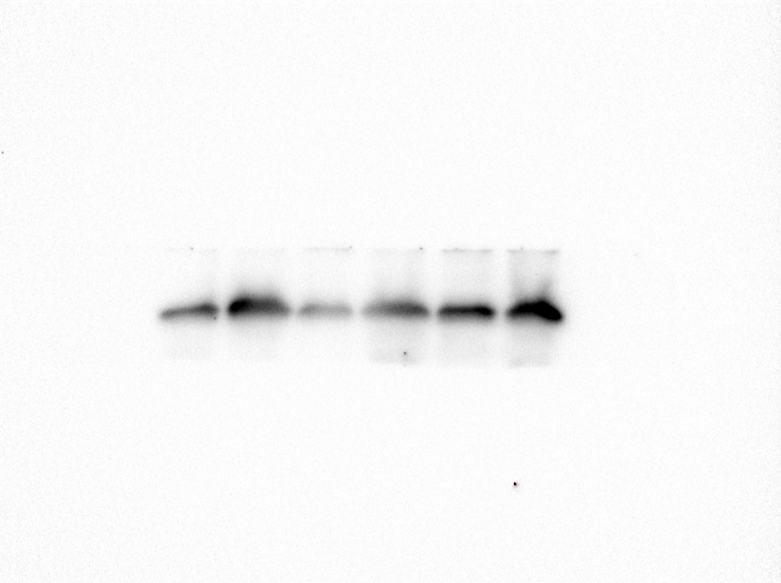


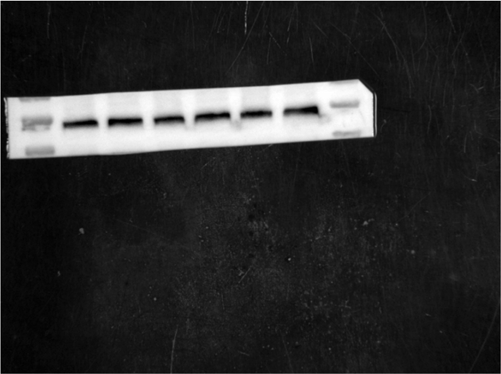
β-tubulin

**Fig. 5** Bcl-2


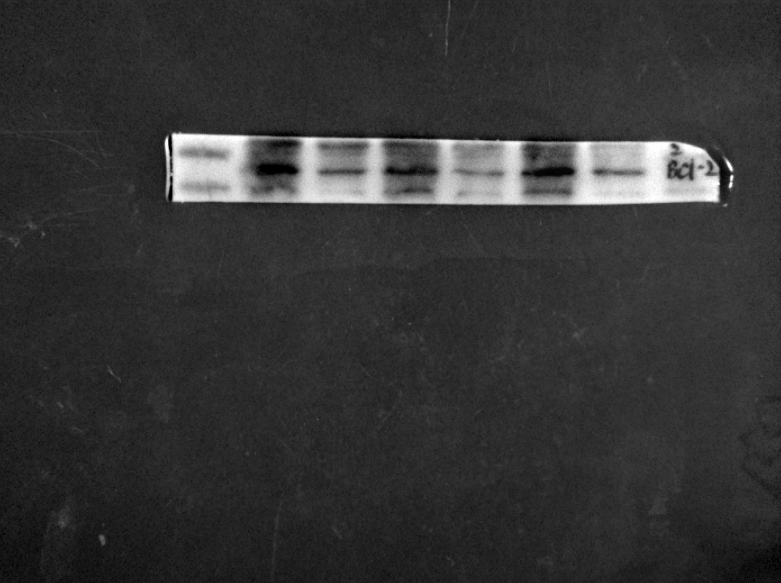


C P C P C P

3#

2#

1#

Bax


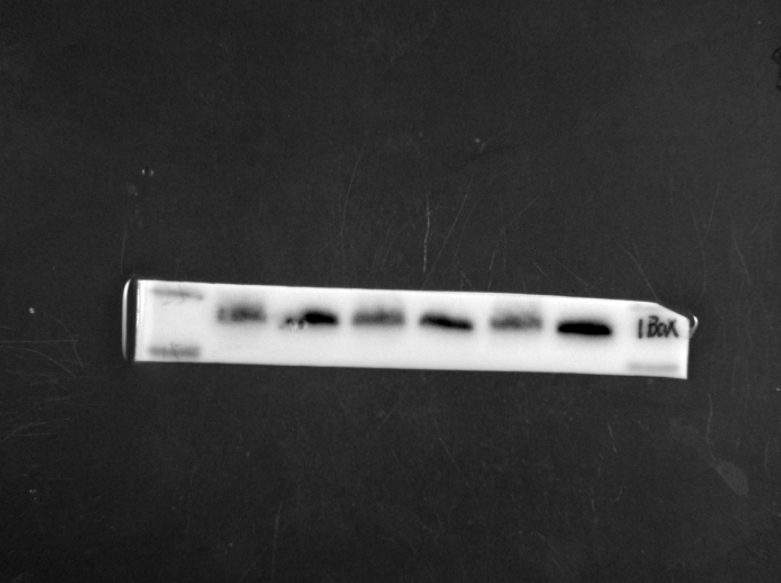


Cleaved-caspase9


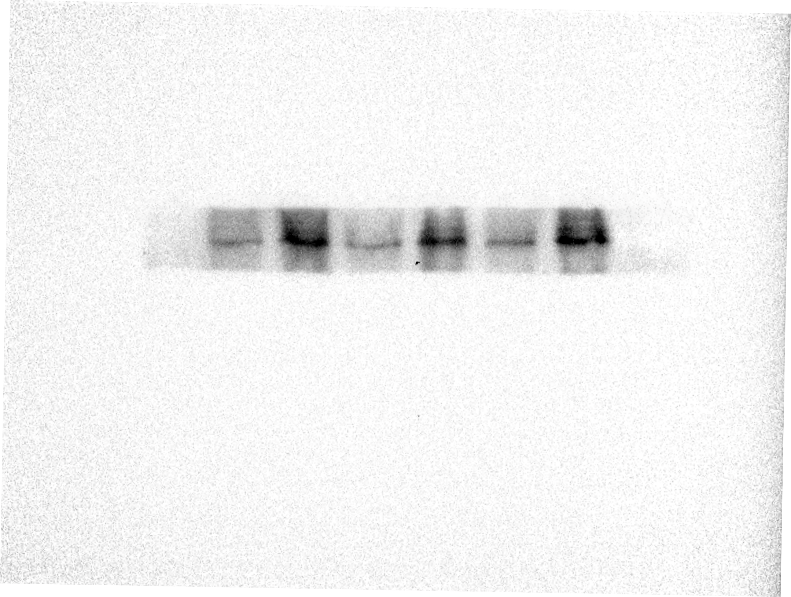


Cleaved-caspase3


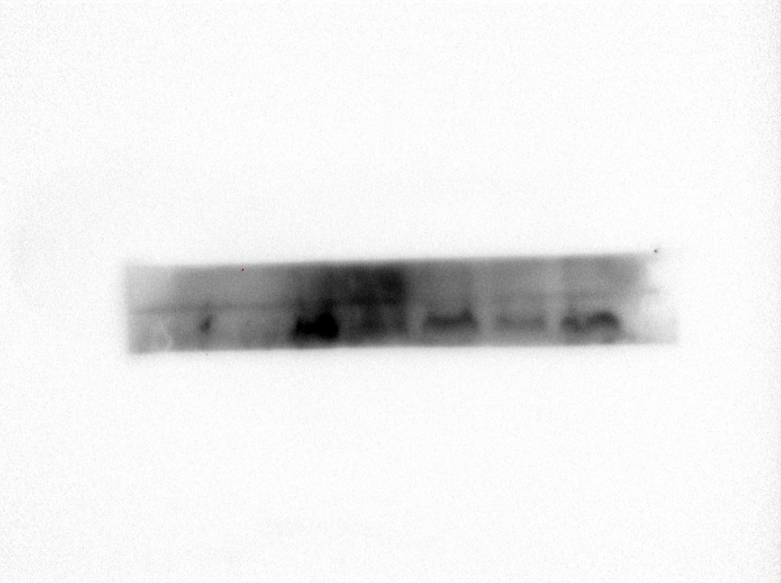


Bid


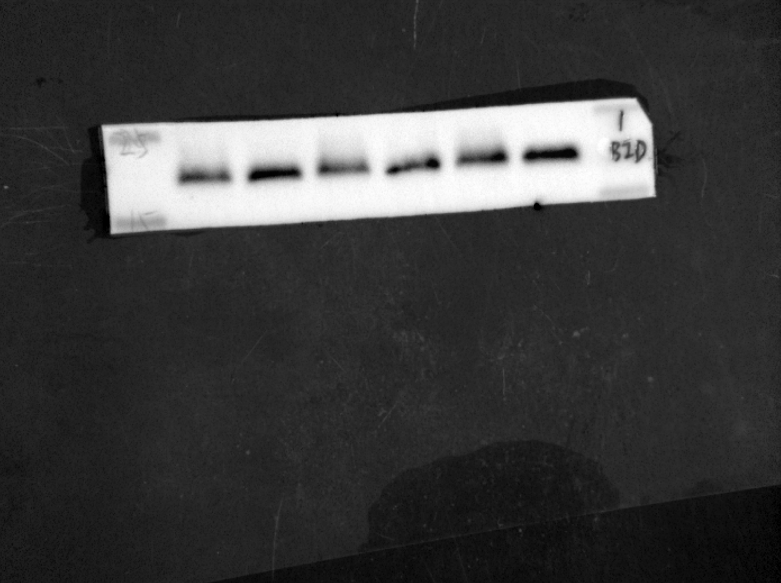


GAPDH


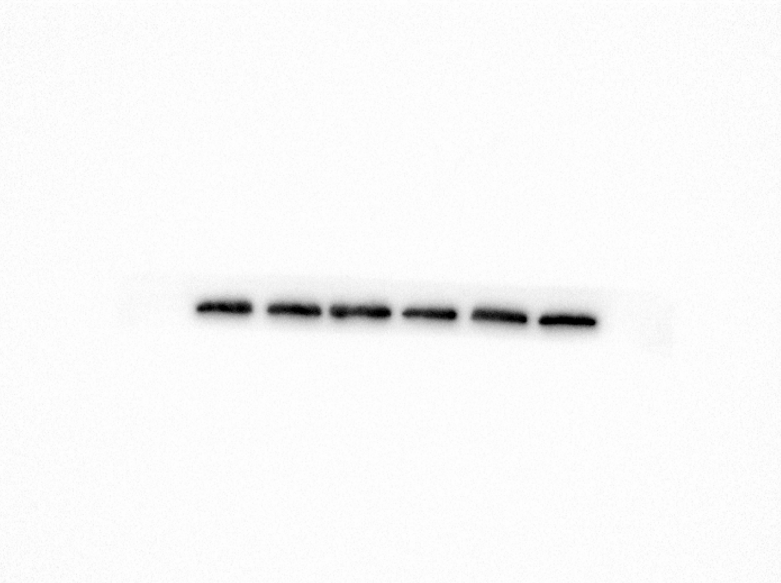


**Fig. 6C** PTPRO


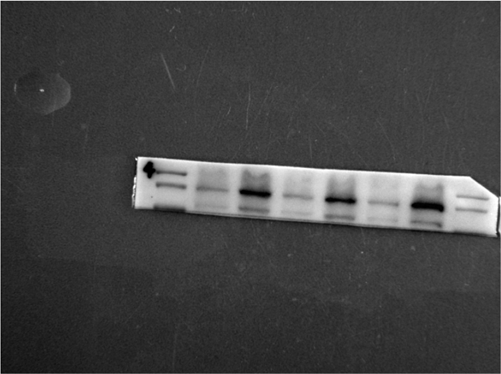


C P

C P C P

H1975

PC9

HCC827

E-cadherin


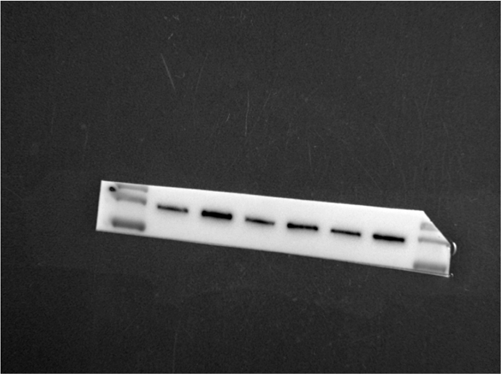


N-cadherin


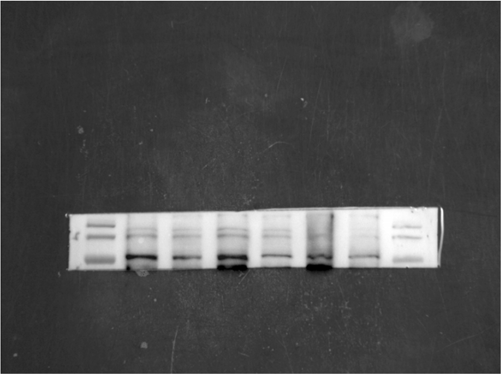


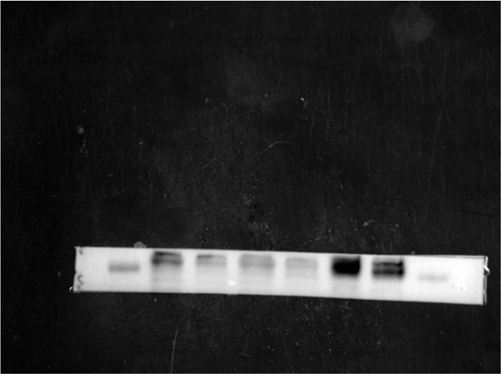
Snail

Gapdh


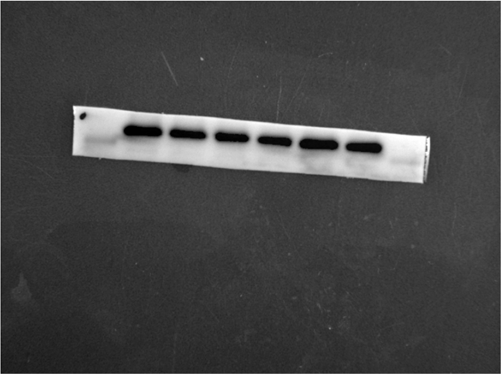


**Fig. 7B** pJAK2


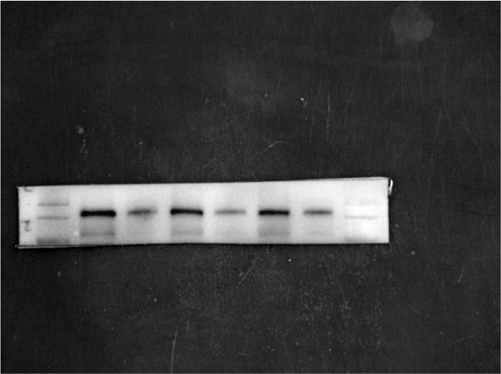


C P

C P C P

H1975

PC9

HCC827


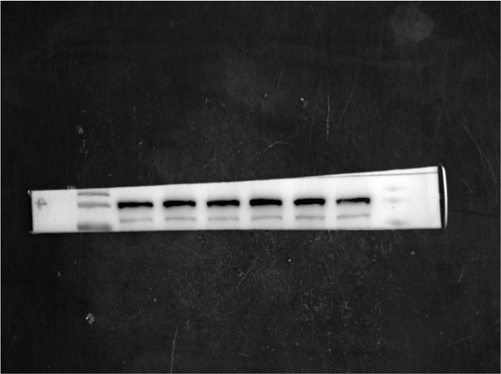
JAK2

p-STAT3


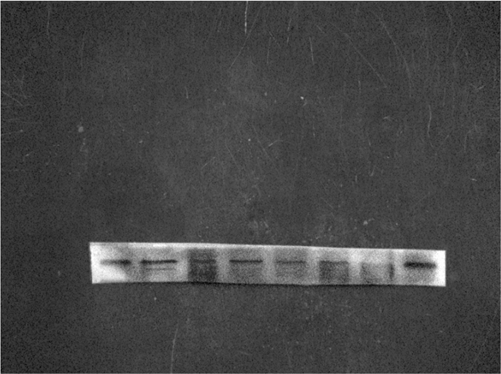


STAT3


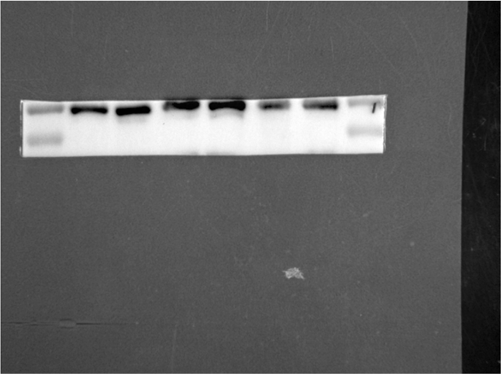


**Fig. 7C** p-JAK2


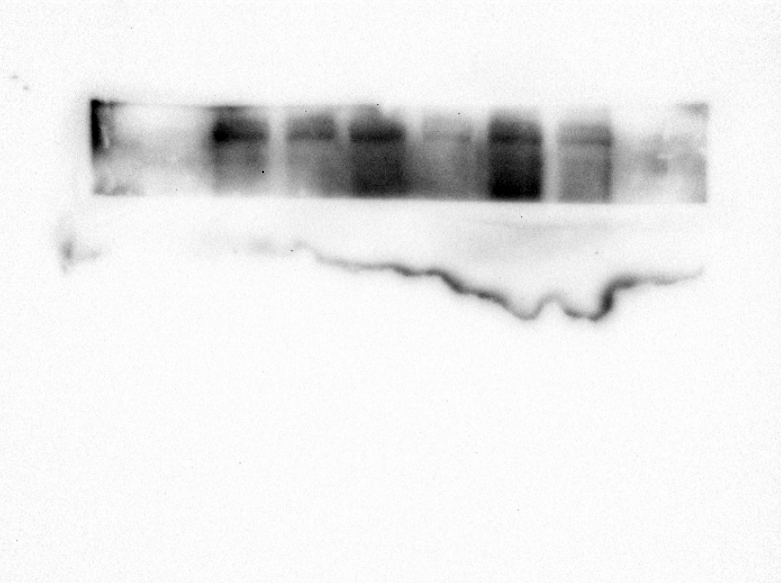


C P C P C P

3#

2#

1#

p-STAT3


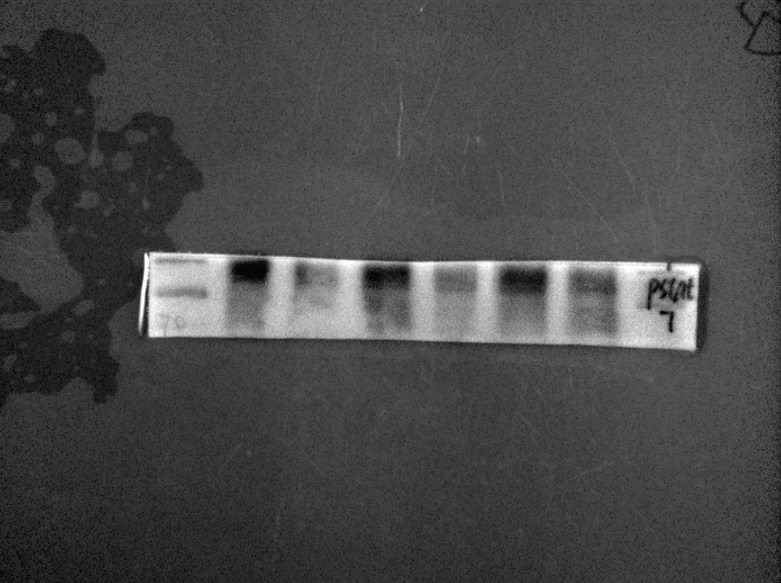


GAPDH


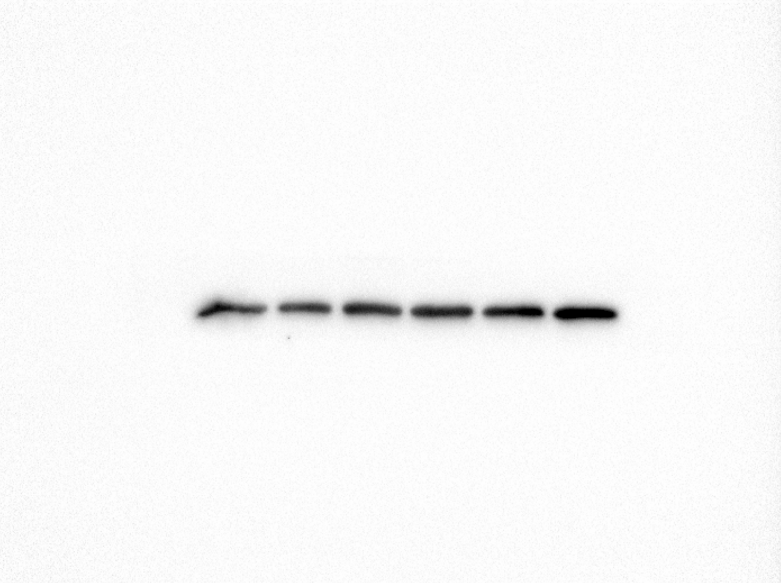


**Fig. 8A**

p-stat3


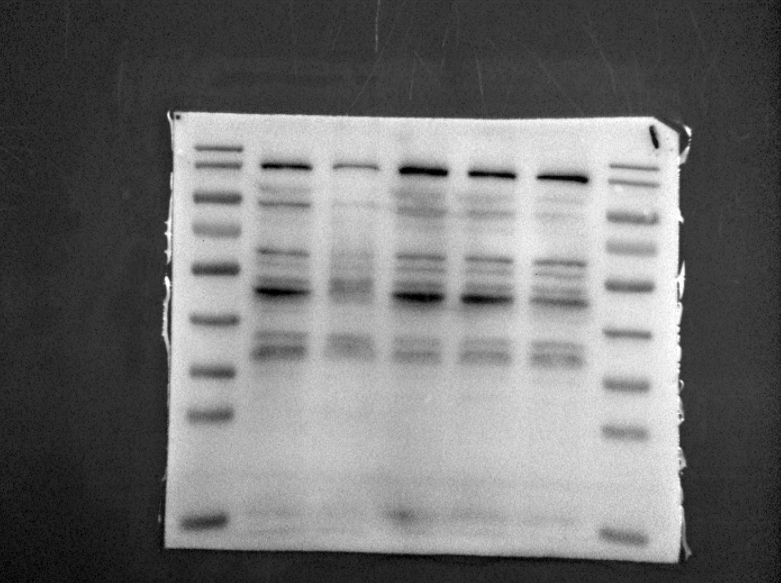


100

70

GAPDH


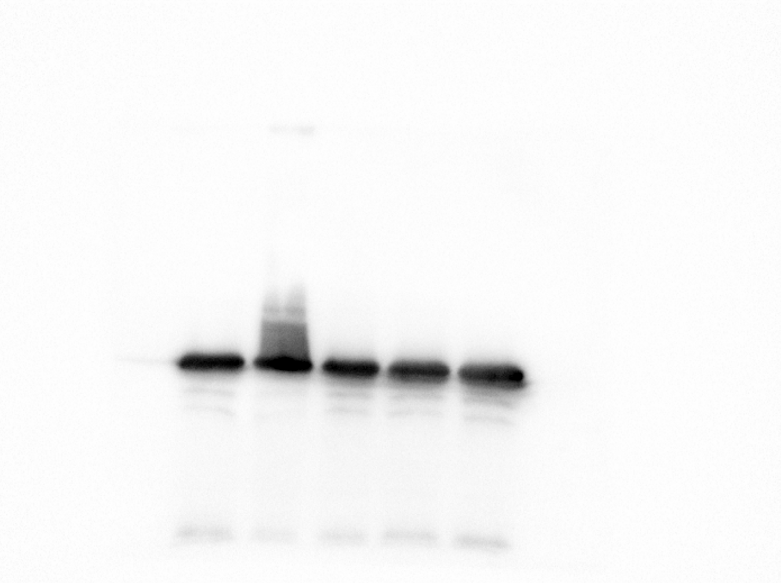

Supplement: Supplementary file 3 — Original Data File [file 41419_2023_6375_MOESM3_ESM.docx]
